# Supplementary material for: Nageotte nodules in human dorsal root ganglia reveal neurodegeneration in diabetic peripheral neuropathy
Source: Nat Commun. 2025 May 5;16:4168. doi: 10.1038/s41467-025-59538-z (PMC12052976; doi:10.1038/s41467-025-59538-z)
Supplement: Supplementary file 2 — Description of Additional Supplementary Files [file 41467_2025_59538_MOESM2_ESM.pdf]

Description of Additional Supplementary Files

File name: Supplementary Data 1
Description: Donor information and medical history. The age, cause of death (COD), dorsal root ganglia used, and medical history summaries (diabetes diagnosis, and keywords related to donor groupings) are provided for all donors.

File name: Supplementary Data 2
Description: Significant Gene Ontology Terms for Nageotte Nodules. Statistically significant gene ontology terms detected at Nageotte nodule barcodes provided by Enrichr. Details on statistics (Fisher's exact test), exact and adjusted p-values are provided in this table.

File name: Supplementary Data 3
Description: Nageotte nodule gene list. All genes (counts per million) detected in Nageotte nodule barcodes across clusters. Neuronal-enriched genes were filtered using a Wilcoxon rank sum test with correction for multiple comparisons. Details can be found in this table.

File name: Supplementary Data 4
Description: Deconvolution of potential mRNA sources in Nageotte nodules.

File name: Supplementary Data 5
Description: RNAscope *in situ* hybridization probes and antibodies list.

File name: Supplementary Data 6
Description: Annotated barcodes. A list of all annotated barcodes for each VISIUM spatial transcriptomic dorsal root ganglion tissue section and instructions on how to import the annotated barcodes into the output VISIUM spatial sequencing cloupe files.

File name: Supplementary Data 7
Description: Spatial RNAsequencing metrics. A full list of quality control metrics such as number of reads and total genes mapped for each dorsal root ganglion tissue section in the VISIUM spatial sequencing experiments.

File name: Supplementary Movie 1
Description: Peripherin-positive axonal fibers sprout throughout the DRG forming Nageotte nodule axon bundles and pericellular nests. 60X z-stack images of peripherin-positive fibers intertwining at a Nageotte nodule and forming pericellular nests (PCNs) around two sensory neurons, one with a visible cell body and another with a misshapen/shrunken soma possibly in a state of dying. The projected z stack image is shown in Supplemental Figure 3C.

File name: Supplementary Movie 2
Description: Nageotte nodule axon bundles originate from dystrophic axons which sprout from local sensory neurons *in situ*. Scan-through of a 60X confocal z-stack image of peripherin (green) and DAPI (blue) staining in a DPN DRG (donor 6). Nageotte nodule fibers, and fibers stemming off of a nearby neuronal cell body were traced as described in Figure 3A.

File name: Supplementary Movie 3
Description: Nageotte nodule axon bundles originate from dystrophic axons which sprout from local sensory neurons *in vitro*. Dissociated human sensory neurons that were

cultured *in vitro* for 3 days display a multipolar phenotype in which multiple axonal branches (peripherin, green) sprout from the neuronal soma, form dystrophic axons (magenta arrows), and intertwine with structures resembling Nageotte nodules. Movie displays the entire z stack of the projected image from Figure 3B.
